# Factors Influencing Help-Seeking Behavior in Patients with Urinary Incontinence: A Single-Center Cross-Sectional Study

**DOI:** 10.3390/medicina61071208

**Published:** 2025-07-02

**Authors:** Mohammed Alshehri, Ebtesam Almajed, Norah Alqntash, Badriyah Abdulaziz AlDejain, Noura Nawar AlQurashi, Nojoud Alamri, Ali AbdelRaheem

**Affiliations:** 1 Department of Urology, King Abdullah Bin Abdulaziz University Hospital, Princess Nourah Bint Abdulrahman University, Riyadh 11671, Saudi Arabia; noaalamri@kaauh.du.sa; 2 Clinical Science Department, College of Medicine, Princess Nourah Bint Abdulrahman University, Riyadh 11671, Saudi Arabia; nhalqntash@gmail.com (N.A.); badriya.aldejain@gmail.com (B.A.A.); 3 College of Medicine, Taif University, Taif 21944, Saudi Arabia; nouraalqurashi5@gmail.com; 4 Department of Urology, Tanta University Hospital, Tanta 31527, Egypt; 5 Department of Urology, King Saud Medical City, Riyadh 11495, Saudi Arabia

**Keywords:** urinary incontinence, facilitators, medical consultation, Saudi Arabia, cross-sectional

## Abstract

*Background and Objectives*: Urinary incontinence (UI) is a prevalent condition that significantly affects quality of life but remains underreported. Understanding the factors that influence patients’ decisions to seek medical consultation is essential for improving care-seeking behavior and ensuring timely intervention. This study aimed to identify the facilitators of seeking medical consultation among individuals with UI in a Saudi secondary care setting. *Materials and Methods*: A cross-sectional study was conducted from December 2024 to April 2025 among adult patients with UI attending urology and urogynecology outpatient clinics at a single tertiary center. Participants completed a structured, self-administered questionnaire that comprised sociodemographic data, the ICIQ-UI SF, and 33 potential motivators for seeking care, categorized into six domains. *Results*: A total of 241 participants were included in the study. The 33-item scale demonstrated excellent internal consistency (Cronbach’s α = 0.945). The most influential domains were daily and physical impact, followed by emotional and psychological factors. Top facilitators included interference with prayers (66.8%), use of pads (62.2%), social limitations (63.9%), frequent clothing changes (64.7%), and fear of worsening symptoms (63.5%). Cultural factors, such as access to same-sex specialists (52.2%), were also prominent. Logistic regression identified age, marital status, and motivators from several domains as significant predictors. Key independent predictors included prayer interference, leakage frequency, and gender-concordant care. *Conclusions*: Help-seeking for UI is influenced by physical, emotional, social, and cultural factors. Enhancing patient education, addressing sociocultural sensitivities, and promoting physician-led discussions foster earlier care-seeking and improve health outcomes in populations with traditionally low treatment uptake.

## 1. Introduction

Urinary incontinence (UI) is a prevalent yet frequently underdiagnosed condition. Although it affects diverse populations, its global prevalence varies widely, ranging from 5% to 70%, and increases with age [1]. Despite its high prevalence, many individuals with UI do not seek medical attention.

Surui Liang et al. conducted a systematic review and meta-analysis to estimate the prevalence of help-seeking behavior among women with UI and identify influencing factors [2]. The review included 41 studies comprising 32,640 women and found that the pooled prevalence of help-seeking behavior was 28%. Subgroup analyses revealed variation in help-seeking rates based on age, UI type, and region. Lower rates were observed among pregnant and younger women, while higher rates were observed among women over 50 and those in Europe. Specifically, the prevalence was 23% among pregnant and maternity women, 27% among menopausal women, 24% among women aged 20–50, 31% among those over 50, 24% in Asia, and 33% in Europe.

The low consultation rate is attributable to various cultural and psychosocial barriers. Embarrassment or shame is consistently identified as the top barrier preventing patients from seeking help [3]. Kinchen et al. conducted a two-stage cross-sectional survey of adult U.S. women to determine the association between UI severity, women’s attitudes towards incontinence, and the impact on quality of life, as well as the reasons behind participants’ decisions to consult a physician about their symptoms [4]. The study sample consisted of 2310, of whom 1970 women had symptoms of UI, and 38% reported that they had initiated a conversation with a physician about incontinence. The study results indicated that there was a significant number of factors associated with treatment seeking, including 3-year symptom duration, a history of a noticeable accident, poorer disease-specific quality of life scores, not feeling embarrassed to speak with a physician about urinary symptoms, interacting with others about UI, and scheduling routine/preventive appointments regularly [4]. Nationally, a Saudi study investigated barriers to care-seeking among 631 participants with UI. Only 21.7% had sought medical consultation. According to the study, the primary reasons for not seeking care included embarrassment (33.9%), the belief that UI is a normal part of aging (18.7%), reluctance to see a male physician (13.2%), and the perception that no treatment was available (12.5%) [5].

Moreover, a study conducted in Germany and Denmark evaluated the predictors and reasons for help-seeking behavior among women with UI and found that the severity and duration of UI significantly predicted help-seeking behavior. Due to their lack of consideration of UI as a problem, women with mild to moderate UI did not seek assistance. In contrast, for severe or very severe UI, German women reported that other illnesses were more important to them, while Danish women reported that they did not have sufficient resources to consult a physician [6]. In another study involving 1231 outpatients from urology and gynecology clinics, 348 women reported UI, of whom 70 sought medical advice. Key motivators included spousal encouragement (22.9%), concerns about maintaining ritual purity for prayer (94.3%), and having educated spouses (30%) [7]. Furthermore, early medical consultation for UI is associated with an improved quality of life, increased self-esteem, and a reduced risk of complications, allowing individuals to anticipate signs of worsening symptoms and implement informed preventive behaviors before complications arise [2].

Collectively, these findings suggest that a complex interplay of personal, clinical, and contextual factors shapes help-seeking behavior. While some patterns, such as severity of symptoms and quality of life impairment, are consistent across regions, others are deeply influenced by cultural norms, social roles, and access to care. In conservative societies like Saudi Arabia, where modesty and religious practices are deeply rooted, other motivators may outweigh social discomfort around discussing urinary symptoms. Despite these insights, a significant gap remains in regional research that explores the facilitators, rather than solely the barriers, of help-seeking behavior for UI, particularly within the Gulf and broader Middle Eastern regions. In Saudi Arabia, in particular, few studies have comprehensively examined how cultural norms, gender roles, healthcare accessibility, and religiosity intersect to influence care-seeking practices. To address this gap, the present study aimed to identify and analyze factors that facilitate help-seeking for UI, informing culturally sensitive interventions and public health strategies that may contribute to reducing the national burden of untreated UI.

## 2. Materials and Methods

### 2.1. Study Design and Population

A cross-sectional study was conducted between December 2024 and April 2025 at a single secondary care center in Saudi Arabia. Patients diagnosed with urinary incontinence (UI) who had sought medical consultation were recruited from the urology and urogynecology outpatient clinics using a non-probability convenience sampling method. While this sampling approach is practical and commonly used in outpatient clinic-based research, it may introduce selection bias and limit the generalizability of the findings beyond similar clinical populations. The inclusion criteria were adults aged 18 years or older who sought medical advice and had been diagnosed with UI. Individuals who had never sought medical care for UI or did not report UI were excluded from the study.

### 2.2. Ethics Statement and Informed Consent

The study received ethical approval from the Institutional Review Board (IRB) at King Abdullah bin Abdulaziz University Hospital (approval number 23-0277). All participants provided informed consent. Responses were collected anonymously, and no identifying personal information was stored.

### 2.3. Study Survey and Data Collection

Data were collected using a structured, self-administered questionnaire distributed in electronic and paper-based formats at the outpatient clinics. The questionnaire comprised three sections, including *(a) sociodemographic and clinical characteristics*, which included age, sex, marital status, education, occupation, income, and comorbid conditions, and (b) a gynecologic history section for female participants, covering gravidity and parity, mode of delivery, menopausal status, and hormone therapy. *(b) Urinary incontinence assessment* used the validated Arabic version of the International Consultation on Incontinence Questionnaire Urinary Incontinence Short Form (ICIQ-UI-SF) [8]. This instrument consists of three scored items that measure frequency, volume of leakage, and impact on quality of life, with a total score ranging from 0 to 21. An additional diagnostic item identifies the context of leakage, allowing classification into stress, urge, or mixed UI. *(c) Treatment-seeking behavior* was explored through the potential motivators, comprising a total of 33 items drawn from Kinchen et al., with a few modifications based on the literature review. We included additional factors identified as facilitators of seeking medical care [4]. The added items focused on cultural, religious, and psychosocial motivators relevant to the local population. All items were reviewed by an expert panel of three urologists and two urogynecologists for clinical and contextual relevance. Motivators were categorized into six domains: (1) impact on daily and physical life, (2) emotional and psychological reflections, (3) severity and frequency of symptoms, (4) encouragement and support, (5) knowledge sources, and (6) personal attempts to manage the condition. Respondents rated each reason on a 5-point Likert scale (“Strongly Disagree” to “Strongly Agree”) and were then asked to select the single most important reason that led them to seek consultation.

The questionnaire, including the newly added motivator items, was developed in English and then translated into Arabic through a standardized forward- and back-translation process by bilingual healthcare professionals. To ensure linguistic accuracy and cultural relevance, the translated version was reviewed by a multidisciplinary expert panel consisting of urologists, urogynecologists, and health education specialists. Minor modifications were made to ensure clarity, cultural sensitivity, and alignment with local terminology. The complete questionnaire was pilot-tested on a group of 15 patients for clarity, linguistic accuracy, and cultural appropriateness, with minor revisions made accordingly.

### 2.4. Statistical Analysis and Data Management

Data were analyzed using IBM SPSS Statistics version 27. Descriptive statistics, including frequencies, percentages, means, and standard deviations, were used to summarize the demographic and clinical characteristics of the participants. The distribution of continuous variables was assessed using the Kolmogorov–Smirnov and Shapiro–Wilk tests. Spearman’s rank correlation was used to assess relationships between ordinal variables for inferential analyses. Group comparisons were performed using non-parametric tests: the Mann–Whitney U test for two-group comparisons and the Kruskal–Wallis test for three or more groups. For the logistic regression models, each domain score was dichotomized into “high” and “low” categories using a median split. Participants with scores equal to or above the median were classified as “high” and those below the median as “low.” A *p*-value < 0.05 was considered statistically significant in all analyses.

Although the questionnaire was comprehensive, no missing data were encountered in the final dataset. This is attributed to the structured format of the survey, close follow-up by research staff during data collection, and the use of required fields in the electronic version. As a result, a complete case analysis was performed without the need for exclusion due to incomplete responses.

## 3. Results

Two hundred forty-one patients with urinary incontinence participated in the study. The questionnaire’s internal consistency was assessed using Cronbach’s alpha. All subscales demonstrated acceptable to excellent reliability, with particularly high consistency in the knowledge sources (α = 0.922) and impact on daily and physical life (α = 0.889) domains. The full 33-item scale yielded an overall Cronbach’s alpha of 0.945, indicating excellent reliability. While this high value supports the internal coherence of the scale, it may also suggest potential redundancy within certain items within specific domains (Table 1).

Table 2 shows the sociodemographic characteristics of the sample. Most of the participants were female (76.3%) with a mean age of 43 ± 12 and an average BMI of 29, suggesting a general trend toward overweight and obesity. The majority were Saudi (95%) and resided in the central region (67.2%), with the remainder distributed across western, eastern, southern, and northern regions. Regarding education, two-thirds held a bachelor’s degree (67.6%), while 11.2% had completed postgraduate studies. Employment status included 36.9% employed and 20.7% housewives. Monthly income levels were diverse, with 40.2% earning less than 5000 SAR. Comorbid chronic illnesses were reported by 61.4% of participants, most commonly diabetes (20.7%) and hypertension (20.3%), with 71% being passive smokers. Regarding outpatient visit history, over half of the participants (53.1%) had visited a doctor between one and four times, while 29% reported having five or more medical consultations.

Table 3 demonstrates the patient-reported frequency, severity, and type characteristics of urinary incontinence according to the ICIQ-UI SF. It revealed varied patterns and severity levels of urinary incontinence among the 241 patients. In terms of frequency, the most reported occurrence was leakage about once a week or less (29%), followed by two to three times a week (25.7%) and once daily (19.9%). A considerable proportion experienced leakage several times daily (20.3%), while 5% reported constant leakage. Regarding the leakage volume, the majority (64.7%) indicated it was small, while 27% described it as moderate and 8.3% as large. The average perceived interference of urinary leakage with daily life was mild, with a mean score of 5 ± 3 on a 0–10 scale. When asked about situations where leakage occurs, the most frequently reported triggers were coughing or sneezing (55.2%) and the need to urinate before reaching the toilet (43.5%). Physical activity or exercise was also a common trigger (30.5%), followed by post-void leakage after dressing (12.1%) and leakage during sleep (11.7%). Fewer participants reported leakage for no apparent reason (7.5%) or continuous leakage (5.4%).

Table 4 displays the determinants and facilitators influencing patients’ decisions to seek medical consultation for urinary incontinence. As indicated by a high percentage of participants who agreed or strongly agreed, the most prominent factors fall within the daily and physical impact, as well as the emotional and psychological domains. A substantial proportion of participants reported that practical disruptions to daily life played a significant role in their decision to seek help. Specifically, 66.8% cited interference with prayers as a motivator. Other frequently reported factors included the need to wear pads or liners (62.2%), interference with social life and gatherings (63.9%), and disruption to daily activities (55.7%). Psychosocial factors were also highly influential, with 64.7% of respondents indicating that being tired from constantly changing clothes motivated them to seek help and 63.5% expressing concern that their UI would worsen over time. Fear of embarrassment (61.4%) and concern that the condition was abnormal (56.9%) were also commonly endorsed. Notably, a significant portion reported concern about underlying serious illness (45.2%) and adverse effects on psychological well-being (49.4%), emphasizing the emotional burden associated with UI.

Furthermore, among symptom-related triggers, experiencing embarrassing accidents (57.3%) and an increased frequency of leakage (37.8%) were notable drivers for seeking consultation. An increase in the amount of leakage (42.4%) and episodes of genital infection (26.1%) were less frequently reported but still contributed to help-seeking behavior. External encouragement was also relevant, with 45.2% reporting spousal support and 43.6% referencing encouragement from friends. Interestingly, doctor-initiated conversations were a motivator for 40.3% of participants. Moreover, awareness of new medications (39.8%), surgical procedures (39.9%), and other treatment modalities, such as Botox or bulking injections (45.7%), motivated a significant subset of participants to seek care. Access to same-sex specialists was important for over half of the respondents (52.2%), reflecting cultural preferences for gender-concordant care. Social media and exposure to awareness campaigns or specialist advertisements were cited by around 40–45% of participants as influential. Finally, personal efforts, such as attempting lifestyle modifications without improvement (55.2%) and the motivation of a special upcoming event (41.5%), also prompted some individuals to seek professional help.

Additionally, participants identified the most important reason that motivated them to seek medical consultation for urinary incontinence. Ten commonly cited motivators emerged. The leading concern was the potential worsening of the condition, reported by 18.3% of respondents. This was followed by the need to begin using panty liners or pads (14.5%) and anxiety about a potentially embarrassing accident (10.4%). Other concerns included perceiving the condition as abnormal (7.9%), experiencing a prior embarrassing incident (7.5%), and fears that leakage signaled a serious health issue (5.8%). Daily activity interference was cited by 5.8% as a key factor in help-seeking. Emotional and social triggers played a more minor but notable role: spousal encouragement and psychological distress each motivated 2.9%, and 2.5% reported that access to a same-sex urologist or urogynecologist influenced their decision.

Table 5 presents the distribution of participants’ responses across six cumulative domains reflecting reasons for seeking medical care. The highest mean score was observed for impact on daily and physical life (mean = 17.8, SD = 6.78), followed by emotional and psychological reflections (mean = 16.9, SD = 7.10), indicating that functional disruption and emotional distress were the most potent motivators. Knowledge sources also showed a notable contribution (mean = 16.2, SD = 9.05), emphasizing the role of awareness in triggering care-seeking. Encouragement and support from others had a moderate impact (mean = 10.5, SD = 5.25), while attempts to manage the condition personally scored the lowest (mean = 4.3, SD = 2.42), suggesting limited reliance on self-management. Overall cumulative scores ranged from 9 to 132, with a mean of 74.0 (SD = 26.36), reflecting considerable variability in participant motivational patterns.

Table 6 shows inferential analysis using Spearman’s rho and Kruskal–Wallis H tests, which revealed several statistically significant associations between sociodemographic variables and the reasons for seeking medical consultation. Age was negatively correlated with emotional and psychological reflections (ρ = −0.212, *p* = 0.001), encouragement and support from others (ρ = −0.192, *p* = 0.003), and the overall reasons score (ρ = −0.196, *p* = 0.002), suggesting that younger participants were more likely to report these factors as motivations. BMI was negatively correlated with all domains, except impact on daily life, with the strongest associations seen for encouragement and support from others (ρ = −0.249, *p* < 0.001) and all reasons (ρ = −0.247, *p* < 0.001). Significant regional differences were observed, with participants from the southern region consistently scoring highest in emotional and psychological reflections, symptom severity, encouragement and support from others, and knowledge sources (*p* < 0.05). Unemployed participants had significantly higher scores across most domains, particularly emotional and psychological reflections (H = 15.6, *p* = 0.004), support from others (H = 15.0, *p* = 0.005), and all reasons (H = 16.3, *p* = 0.003).

The binary logistic regression findings are summarized in Table 7, highlighting key sociodemographic predictors across six domains of help-seeking behavior for urinary incontinence. In the domain of daily and physical life, younger individuals were more likely to report a higher impact (AOR = 0.975, *p* = 0.030), indicating greater disruption in their daily functioning. Widowed participants had significantly higher odds of reporting impact than singles (AOR = 8.371, *p* = 0.022), suggesting a substantial burden in this group. For the emotional and psychological reflections domain, younger age again predicted greater emotional concern (AOR = 0.972, *p* = 0.030). Employees (AOR = 0.316, *p* = 0.029) and housewives (AOR = 0.227, *p* = 0.004) were significantly less likely than students to report emotional motivations, indicating a 68% and 77% reduction in odds, respectively. At the same time, participants with a monthly income of 5000–10,000 SAR were more likely to report such motivations (AOR = 2.474, *p* = 0.020), highlighting income-related variation in perceived emotional burden. In the domain of symptom severity and frequency, married participants (AOR = 0.434, *p* = 0.046) and divorced participants (AOR = 0.231, *p* = 0.011) were less likely than singles to report severe symptoms. Housewives had markedly higher odds of reporting symptom severity than students (AOR = 6.381, *p* = 0.020), indicating they were over six times more likely to experience or acknowledge bothersome symptoms. The encouragement and support from other domains showed lower odds of support among obese individuals (AOR = 0.136, *p* = 0.010), males (AOR = 0.367, *p* = 0.019), and bachelor’s degree holders (AOR = 0.381, *p* = 0.045). In contrast, non-Saudi nationals (AOR = 9.330, *p* = 0.011), unemployed individuals (AOR = 5.841, *p* = 0.010), and those with higher incomes (AORs = 2.878–3.700) were more likely to report receiving support, indicating stronger social encouragement within these groups. Regarding knowledge sources, younger age showed a borderline association with higher knowledge-related motivations (AOR = 0.978, *p* = 0.050), suggesting that older individuals may have slightly less exposure to or interest in health information. BMI demonstrated a clear inverse trend, with overweight (AOR = 0.254, *p* = 0.052) and obese individuals (AOR = 0.147, *p* = 0.008) significantly less likely to report informational motivations than underweight individuals. Additionally, participants earning 5000–10,000 SAR were more likely to report knowledge-based reasons for seeking care than those earning less than 5000 SAR (AOR = 2.123, *p* = 0.019). Lastly, in the domain of attempts to manage the condition personally, younger participants were more likely to report managing their condition independently (AOR = 0.968, *p* = 0.029), with increasing age associated with a modest decline in self-care. Compared to underweight individuals, overweight (AOR = 0.153, *p* = 0.015) and obese participants (AOR = 0.164, *p* = 0.021) had significantly lower odds of self-management. Additionally, those residing in the central region were less likely to engage in self-care compared to participants from the western region (AOR = 0.347, *p* = 0.039), suggesting possible regional differences in personal health practices.

## 4. Discussion

Urinary incontinence (UI) is a common condition with significant physical, emotional, and social consequences, yet the factors influencing patients’ decisions to seek medical consultation remain underexplored in the Saudi population. Despite its considerable impact on quality of life, the existing literature in the region lacks a comprehensive examination of the multifaceted reasons that drive patients to seek medical help. This study was, therefore, necessary to fill a critical gap in understanding the sociodemographic, psychological, and behavioral factors influencing healthcare-seeking behavior among UI patients in Saudi Arabia. The results revealed that the most influential domains were daily and physical impact, followed by the emotional and psychological domains. The most endorsed facilitators were interference with prayers, wearing pads or liners, social reasons, and emotional factors, such as the need for frequent clothing changes and concern about the condition worsening. Cultural factors, such as access to same-sex specialists and embarrassment-related barriers, were similarly prominent. While knowledge of treatment options played a role, awareness levels were relatively low across the sample.

UI affects daily functioning, particularly in areas such as physical activity and social engagement. A study of 1446 women found that mixed incontinence and symptom severity were linked to reduced physical activity, alongside factors like age, BMI, pelvic pain, and income [9]. Similarly, a systematic review confirmed a broad decline in quality of life due to UI [10], and a mixed-methods study highlighted that embarrassment and lack of support further limit participation in exercise [11]. Consistent with these findings, 55% of our participants reported interference with daily tasks, 54% with physical activity, 52.7% with work, 66% with prayers, and 63% with social events. This widespread impact highlights the need for multidisciplinary strategies that address quality of life beyond symptom control.

Demographic factors, including age, body mass index (BMI), marital status, and employment status, showed notable associations with help-seeking behavior and symptom burden among individuals with UI. A younger age was significantly associated with higher scores in the emotional, informational, and overall help-seeking domains, suggesting a greater awareness or willingness to seek care. This contrasts with the findings of a Korean study, which showed that both the prevalence of UI and help-seeking behavior increased with age [12]. Moreover, a lower BMI was linked to higher help-seeking scores across most domains, particularly encouragement and support from others. In contrast, a U.S. study found that women with a BMI >25 kg/m^2^ experienced greater distress and were more likely to seek evaluation [13], possibly reflecting cultural differences. Marital status showed a marginal association with help-seeking, with single individuals tending to report higher scores in the emotional and severity domains. This may reflect differences in perceived social support or coping strategies. However, it is not possible to determine whether this association reflects greater emotional distress or simply a differing reliance on healthcare services due to being unpartnered. As this remains speculative, future qualitative research is warranted to unpack the specific experiences and needs of single individuals with UI. This differs from a study conducted in Riyadh, which reported a higher prevalence of UI among married women, likely reflecting childbirth-related risk factors [14]. Therefore, prevalence does not always translate into help-seeking behavior, as engagement may be influenced by social support, stigma, or health priorities. Furthermore, employment status was also influential, with unemployed participants reporting the highest scores across nearly all help-seeking domains, particularly emotional and psychological reflections, support from others, and overall reasons for seeking care. Employees were less likely to report emotional triggers, suggesting that occupational engagement may buffer distress or reduce the perceived need for help. This aligns with a study of working women where only 36% reported their UI to a healthcare provider despite its prevalence, underscoring the impact of employment on help-seeking behavior [15]. These findings underscore the need for targeted health campaigns and intervention strategies tailored to diverse social contexts, relationship statuses, and employment backgrounds.

In our study, participants from the southern region exhibited higher motivator scores across several domains. Although our cross-sectional design precludes causal conclusions, this trend may reflect regional disparities in healthcare accessibility and health literacy, as well as cultural and religious practices that can amplify the emotional and ritual disruptions caused by UI. Specifically, healthcare access is often more limited in southern communities compared to larger urban centers, potentially creating a greater sense of urgency to seek care once symptoms are recognized as problematic.

This study revealed limited awareness of UI treatment options, with fewer than half of the participants familiar with interventions such as medications, surgery, or electrical stimulation. Nonetheless, over 40% reported that social media and advertisements influenced their decision to seek care. In a randomized trial, social media outreach was found to be more effective than search engine ads in raising awareness of stress UI treatments [16]. Interestingly, another study reported that while 51.8% of women had adequate knowledge and 98.7% held positive attitudes, only 29.5% pursued treatment, and 16.4% engaged in prevention [17]. The reasons behind this gap between awareness and treatment-seeking behavior remain unclear, underscoring the need for targeted educational efforts to enhance awareness and promote the transition from knowledge to action.

Social support plays a crucial role in helping individuals seek assistance for urinary incontinence. A qualitative study from Iran identified encouragement from family, friends, and healthcare providers as a key facilitator, while lack of support was a barrier [18]. Furthermore, gender-based differences have been documented. A Turkish study found that only 34.0% of men sought medical care compared to 50.6% of women, suggesting stigma and underreporting among males [19]. Additionally, higher education has been linked to increased help-seeking, likely due to better health literacy and access to care [2]. In line with this, our study found that encouragement and support had a moderate influence on medical consultation, with fewer than half of the participants receiving support from spouses, family, or friends. Notably, support was significantly lower among male participants, highlighting a potential gender disparity in perceived encouragement. This finding aligns with previous research suggesting that social norms, embarrassment, and fear of judgment may discourage men from discussing sensitive health issues, such as urinary symptoms. Similarly, support was lower among obese individuals, potentially reflecting weight-related stigma and lower healthcare engagement.

Income played a notable role, with individuals earning 5000–10,000 SAR being more likely to seek help due to emotional motivators and showing slightly higher awareness of treatment options. While this might suggest a relationship between middle-income status and health engagement, this interpretation should be approached with caution, as income may also reflect differences in healthcare exposure or social pressures rather than a direct causal effect. These findings highlight how region and socioeconomic status shape perceptions and healthcare-seeking behaviors. These results are supported by previous studies from Saudi Arabia. A study in the eastern region reported that women with lower education and unemployment experienced poorer quality of life due to UI, highlighting the role of socioeconomic disadvantage in condition management [20]. Additionally, a systematic review found wide regional variation in stress UI prevalence (3.3–50%) across the country [21], likely reflecting differences in healthcare access, cultural norms, and economic conditions. This highlights the necessity for region-specific strategies to address local needs and mitigate disparities in care.

Motivations tied to events, such as weddings or social gatherings, influenced only 41.5% of participants, indicating a limited impact on long-term help-seeking behavior. Similarly, other findings indicate that individuals often rely on self-directed strategies, such as fluid restriction and absorbent products, which are typically inadequate and may delay proper care [19]. In contrast, a randomized trial demonstrated that structured self-monitoring, including fluid and caffeine management, pelvic floor exercises, and scheduled voiding, significantly reduced urine loss and improved quality of life [22]. This difference may be attributed to the guided, evidence-based nature of the intervention, as opposed to the unstructured and often misinformed approaches commonly used by the public.

This study provides a comprehensive understanding of the factors influencing help-seeking behavior in urinary incontinence, highlighting the emotional burden, life disruption, social support, and informational awareness. These insights support the need for culturally tailored education and community interventions. Community-based strategies, such as gender-specific awareness campaigns, outreach through male-focused community settings, and inclusive messaging in primary care, may help normalize care-seeking behaviors, reduce stigma, and improve access to support. A particularly noteworthy finding was that a substantial proportion of participants cited a doctor-initiated conversation as a motivator for seeking care. This highlights the pivotal role that primary care physicians and general practitioners can play by incorporating UI screening questions into routine check-ups, which may facilitate earlier detection and management. Public health initiatives could also benefit from training healthcare providers to recognize cues, initiate sensitive conversations, and refer appropriately, especially in settings where stigma remains a significant barrier.

Additionally, the high internal consistency observed in our framework suggests potential conceptual overlap among some items. Future research could explore refining or shortening the instrument to enhance efficiency without compromising validity. Future studies should also examine outcomes of targeted interventions, provider readiness, and patient experiences through qualitative methods.

Despite its strengths, this study has several limitations that should be acknowledged. First, the cross-sectional design limits the ability to establish causal relationships between demographic characteristics and help-seeking behavior. Second, the reliance on self-reported data may introduce recall or response bias, particularly in sensitive areas such as emotional distress, religious practices, and urinary symptoms. Third, although the sample was geographically diverse, specific subgroups, particularly males and individuals with lower incomes, were underrepresented, and the overall sample was predominantly female and highly educated. These demographic characteristics may have influenced the observed patterns of help-seeking, particularly in domains related to emotional burden and informational awareness. They may limit the generalizability of the findings to broader or more varied populations. Moreover, the influence of healthcare access and provider availability, though likely important, was not directly assessed. Finally, the study did not include individuals who have never sought care for UI, a population that may face greater stigma, knowledge gaps, or access challenges.

## 5. Conclusions

This study highlights the multifaceted interplay of cultural, emotional, and practical factors influencing help-seeking behavior among individuals with urinary incontinence. Key facilitators included emotional distress, social encouragement, informational awareness, and disruptions to daily life. Demographic variables, including younger age, lower BMI, single status, and middle income, were significantly associated with greater motivation to seek care, particularly in the emotional and knowledge domains. These findings underscore the importance of culturally sensitive interventions that leverage established facilitators, mitigate stigma, and facilitate timely medical consultation. Patient education, gender-concordant care access, and proactive physician engagement may enhance early intervention and improve clinical outcomes.

## Figures and Tables

**Table 1:** Internal consistency of the questionnaire.

Domains	Cronbach’s Alpha	Num. of Items
Impact on Daily and Physical Life	0.889	7
Emotional and Psychological Reflections	0.879	7
Severity and Frequency of Symptoms	0.745	4
Encouragement and Support from Others	0.865	5
Knowledge Sources	0.922	8
Attempts to Manage the Condition Personally	0.790	2
Total	0.945	33

**Table 2:** Sociodemographic characteristics of the sample (*n* = 241).

Variables	Category	*n* (%)	Mean ± SD
Age (Years)			43 ± 12
Weight (kg)			71 ± 18
Height (cm)			161 ± 14
BMI (kg/m^2^)			29 ± 29
BMI Category	Underweight	15 (6.2)	
	Normal	80 (33.2)	
	Overweight	75 (31.1)	
	Obese	71 (29.5)	
Gender	Female	184 (76.3)	
	Male	57 (23.7)	
Nationality	Saudi	229 (95)	
	Non-Saudi	12 (5)	
Current Residency	Western region	27 (11.2)	
	Central region	162 (67.2)	
	Eastern region	28 (11.6)	
	North region	8 (3.3)	
	South region	16 (6.6)	
Marital Status	Single	59 (24.5)	
	Married	141 (58.5)	
	Divorced	31 (12.9)	
	Widowed	10 (4.1)	
Highest Educational Level	School	51 (21.2)	
	Bachelor’s degree	163 (67.6)	
	Postgraduate degree (Master’s, PhD, etc.)	27 (11.2)	
Occupation Status	Student	33 (13.7)	
	Employee	89 (36.9)	
	Retired	38 (15.8)	
	Unemployed	31 (12.9)	
	Housewife	50 (20.7)	
Average Monthly Income (SAR)	Less than 5000 SAR	97 (40.2)	
	5000–10,000 SAR	87 (36.1)	
	More than 10,000 SAR	57 (23.7)	
Chronic Diseases	Diabetes mellitus	50 (20.7)	
	Hypertension	49 (20.3)	
	Asthma	32 (13.3)	
	Cardiovascular diseases	24 (10)	
	Chronic constipation	37 (15.4)	
	None	93 (38.6)	
	Other	51 (21.2)	
Medical Consultation Visits over the Past Year	None	43 (17.8)	
	1–4 visits	128 (53.1)	
	5+	70 (29)	
Smoking Status	Yes	32 (13.3)	
	Previous smoker	36 (14.9)	
	Not a smoker	2 (0.8)	
	Passive smoker	171 (71)	
Gynecological Information, *n* = 184			
Gravidity			4 ± 3
Parity			3 ± 2
Mode of Delivery	Never	38 (20.7)	
	Vaginal delivery	104 (56.5)	
	Cesarean section	9 (4.9)	
	Both	33 (17.9)	
Menstrual History	Regular period	95 (51.6)	
	Irregular period	47 (25.5)	
	Postmenopausal	42 (22.8)	

**Table 3:** Patient-reported frequency, severity, and type characteristics of urinary incontinence according to the ICIQ-UI SF (*n* = 241).

Variables	Category	*n* (%)	Mean ± SD
Frequency of Urine Leakage	About once a week or less often	70 (29)	
	Two or three times a week	62 (25.7)	
	Once a day	48 (19.9)	
	Several times a day	49 (20.3)	
	All the time	12 (5)	
Amount of Urine Leak	A small amount	156 (64.7)	
	A moderate amount	65 (27)	
	A large amount	20 (8.3)	
Impact of Leakage on Daily Life (0–10 Scale)			5 ± 3
Situation/Time of Leakage	Leaks before reaching the toilet	104 (43.5)	
	Leaks when coughing or sneezing	132 (55.2)	
	Leaks during sleep	28 (11.7)	
	Leaks during physical activity/exercise	73 (30.5)	
	Leaks after finishing urination and dressing	29 (12.1)	
	Leaks for no apparent reason	18 (7.5)	
	Leaks all the time	13 (5.4)	

**Table 4:** Determinants and facilitators for seeking medical consultation for urinary incontinence.

Domain	Key Item/Variable	Agree/Strongly Agree (%)	Mean (SD)
Daily and Physical Impact	Wearing pads/liners	62.2	2.7 (1.3)
	Interference with daily activities	55.7	2.5 (1.2)
	Interference with physical activities	54.0	2.4 (1.3)
	Interference with work activities	52.7	2.4 (1.3)
	Interference with sexual activities	46.0	2.3 (1.3)
	Interference with prayers (religious cause)	66.8	2.8 (1.2)
	Interference with social life/attending gatherings and events	63.9	2.7 (1.3)
Emotional/Psychological	Concerned that the condition is not normal	56.9	2.4 (1.3)
	Fear of embarrassment	61.4	2.6 (1.2)
	Worry UI may worsen	63.5	2.6 (1.3)
	Worry about an underlying serious illness	45.2	2.2 (1.4)
	Odor concern	51.4	2.2 (1.4)
	Psychological well-being affected	49.4	2.3 (1.4)
	Tired of changing clothes constantly	64.7	2.6 (1.3)
Symptom Severity	The frequency of leakage increased	37.8	2.1 (1.3)
	Embarrassing accidents	57.3	2.5 (1.2)
	Leakage volume increased	42.4	2.1 (1.3)
	Genital infection	26.1	1.6 (1.3)
Support and Encouragement	Spouse encouragement	45.2	2.1 (1.3)
	Family encouragement	36.9	2.0 (1.3)
	Friends’ encouragement	43.6	2.1 (1.3)
	The doctor initiated the conversation	40.3	2.1 (1.3)
	Less embarrassment after discussing with others	42.8	2.1 (1.3)
Knowledge Sources	Aware of new medications	39.8	2.0 (1.4)
	Aware of new surgical procedures	39.9	2.0 (1.4)
	Aware of electrical stimulation	41.1	1.9 (1.4)
	Aware of Botox/bulking injections	45.7	2.1 (1.4)
	Attended awareness campaigns	36.5	1.9 (1.4)
	Social media motivated care-seeking	45.6	2.1 (1.4)
	Saw specialist advertisements	42.8	2.0 (1.4)
	Access to a same-sex urologist/urogynecologist	52.2	2.3 (1.4)
Personal Efforts	Special event motivated care-seeking	41.5	1.9 (1.3)
	Tried lifestyle modifications, but no improvement	55.2	2.4 (1.3)

**Table 5:** Statistical summary of reasons for seeking medical consultation variables.

Reasons	Mean	Median	Standard Deviation	Minimum	Maximum
Impact on Daily and Physical Life	17.8	18	6.78	0	28
Emotional and Psychological Reflections	16.9	18	7.10	0	28
Severity and Frequency of Symptoms	8.3	9	3.84	0	16
Encouragement and Support from Others	10.5	11	5.25	0	20
Knowledge Sources	16.2	17	9.05	0	32
Attempts to Manage the Condition Personally	4.3	5	2.42	0	8
All Reasons	74.0	77	26.36	9	132

**Table 6:** Inferential statistical summary for correlation between significant demographic variables and reasons for seeking medical consultation variables.

Independent Variable	Dependent Variables	Type of Test	Value	*p*-Value	Highest Mean Rank (Category)
Age	Impact on Daily and Physical Life	Spearman’s rho	−0.106	0.101	
	Emotional and Psychological Reflections		−0.212 **	0.001	
	Severity and Frequency of Symptoms		−0.102	0.115	
	Encouragement and Support from Others		−0.192 **	0.003	
	Knowledge Sources		−0.127 *	0.049	
	Attempts to Manage the Condition Personally		−0.109	0.091	
	All Reasons		−0.196 **	0.002	
BMI	Impact on Daily and Physical Life	Spearman’s rho	−0.016	0.805	
	Emotional and Psychological Reflections		−0.225 **	0.000	
	Severity and Frequency of Symptoms		−0.221 **	0.001	
	Encouragement and Support from Others		−0.249 **	0.000	
	Knowledge Sources		−0.241 **	0.000	
	Attempts to Manage the Condition Personally		−0.168 **	0.009	
	All Reasons		−0.247 **	0.000	
Current Residency	Impact on Daily and Physical Life	Kruskal–Wallis H	H(3) = 2.9	0.568	South (136.69)
	Emotional and Psychological Reflections		H(3) = 12.8	0.012	South (152.34)
	Severity and Frequency of Symptoms		H(3) = 12.5	0.014	South (163.41)
	Encouragement and Support from Others		H(3) = 13.2	0.010	South (165.59)
	Knowledge Sources		H(3) = 13.7	0.008	South (169.63)
	Attempts to Manage the Condition Personally		H(3) = 9.6	0.047	South (150.5)
	All Reasons		H(3) = 17.1	0.002	South (170.5)
Marital Status	Impact on Daily and Physical Life	Kruskal–Wallis H	H(3)= 10.9	0.012	Widowed (144.60)
	Emotional and Psychological Reflections		H(3) = 20.9	0.000	Single (143.73)
	Severity and Frequency of Symptoms		H(3) = 12.9	0.005	Single (145.05)
	Encouragement and Support from Others		H(3) = 10.3	0.015	Single (134.31)
	Knowledge Sources		H(3) = 1.5	0.678	Single (127.47)
	Attempts to Manage the Condition Personally		H(3) = 11.8	0.008	Widowed (139.40)
	All Reasons		H(3) = 14.2	0.003	Single (137.04)
Occupation Status	Impact on Daily and Physical Life	Kruskal–Wallis H	H(4) = 9.5	0.050	Unemployed (134.73)
	Emotional and Psychological Reflections		H(4) = 15.6	0.004	Unemployed (151.42)
	Severity and Frequency of Symptoms		H(4) = 9.1	0.057	Unemployed (138.85)
	Encouragement and Support from Others		H(4) = 15.0	0.005	Unemployed (156.15)
	Knowledge Sources		H(4 )= 10.3	0.035	Unemployed (141.06)
	Attempts to Manage the Condition Personally		H(4) = 8.0	0.090	Unemployed (138.88)
	All Reasons		H(4) = 16.3	0.003	Unemployed (150.97)

* at 0.05; ** at 0.01.

**Table 7:** Binary logistic regression between selected sociodemographic variables and reasons for seeking medical consultation domains.

Variables	Univariate Model		Multivariate Model	
	Crude OR (95% CI)	*p*-Value	Adjusted OR (95% CI)	*p*-Value
a. Impact on Daily and Physical Life				
Age	0.981 [0.961–1.002]	0.077	0.975 [0.951–0.999]	0.039
Single	Reference		Reference	
Married	1.412 [0.765–2.607]	0.270	2.127 [0.993–4.556]	0.052
Divorced	0.430 [0.173–1.069]	0.069	0.611 [0.217–1.720]	0.351
Widowed	3.613 [0.707–18.469]	0.123	8.371 [1.350–51.916]	0.022
b. Emotional and Psychological Reflections				
Age	0.971 [0.950–0.991]	0.006	0.972 [0.947–0.997]	0.030
Student	Reference		Reference	
Employee	0.425 [0.182–0.995]	0.049	0.316 [0.113–0.890]	0.029
Housewife	0.224 [0.087–0.576]	0.002	0.227 [0.082–0.628]	0.004
Western Region	Reference		Reference	
South Region	5.600 [1.060–29.592]	0.043	4.448 [0.756–26.187]	0.099
Less than 5000 SAR	Reference		Reference	
5000–10,000 SAR	1.828 [1.013–3.300]	0.045	2.474 [1.157–5.291]	0.020
c. Severity and Frequency of Symptoms				
Student	Reference		Reference	
Housewife	0.366 [0.148–0.905]	0.030	6.381 [1.340–30.373]	0.020
Single	Reference		Reference	
Married	0.375 [0.197–0.716]	0.003	0.434 [0.191–0.985]	0.046
Divorced	0.180 [0.069–0.466]	<0.001	0.231 [0.075–0.712]	0.011
d. Encouragement and Support from Others				
Age	0.972 [0.952–0.993]	0.008	0.987 (0.958–1.016)	0.371
Underweight	Reference		Reference	
Obese	0.196 [0.060–0.646]	0.007	0.136 [0.030–0.615]	0.010
Saudi	Reference		Reference	
Non-Saudi	5.223 [1.120–24.367]	0.035	9.330 [1.680–51.813]	0.011
Female	Reference		Reference	
Male	0.924 [0.510–1.675]	0.796	0.367 [0.159–0.848]	0.019
Student	Reference		Reference	
Unemployed	3.472 [1.128–10.685]	0.030	5.841 [1.514–22.536]	0.010
School	Reference		Reference	
Bachelor’s Degree	1.133 [0.604–2.126]	0.697	0.381 [0.149–0.979]	0.045
Less than 5000 SAR	Reference		Reference	
5000–10,000 SAR	1.365 [0.763–2.439]	0.294	2.878 [1.719–7.029]	0.020
More than 10,000 SAR	1.070 [0.556–2.060]	0.838	3.700 [1.273–10.755]	0.016
e. Knowledge Sources				
Age	0.972 [0.951–0.992]	0.007	0.978 (0.956–1.000)	0.050
Underweight	Reference		Reference	
Overweight	0.243 [0.064–0.933]	0.039	0.254 [0.064–1.012]	0.052
Obese	0.153 [0.040–0.593]	0.007	0.147 [0.036–0.600]	0.008
Less than 5000 SAR	Reference		Reference	
5000–10,000 SAR	1.647 [0.917–2.957]	0.095	2.123 [1.134–3.976]	0.019
f. Attempts to Manage the Condition Personally				
Age	0.969 [0.949–0.990]	0.004	0.968 (0.941–0.997)	0.029
Underweight	Reference		Reference	
Overweight	0.302 [0.088–1.033]	0.056	0.153 [0.034–0.693]	0.015
Obese	0.316 [0.092–1.087]	0.068	0.164 [0.035–0.757]	0.021
Western Region	Reference		Reference	
Central Region	0.507 [0.219–1.175]	0.113	0.347 [0.127–0.948]	0.039

## Data Availability

Data from this study is available for sharing upon request.
